# Analysis of Multiphase Flow and Heat and Mass Transfer for Ammonium Chloride Crystallization of the High-Pressure Heat Exchanger in Hydrogenation Unit

**DOI:** 10.3390/ma14247754

**Published:** 2021-12-15

**Authors:** Jianwen Zhang, Yahui Zhao, Yan Li, Fan Zhang

**Affiliations:** 1 College of Mechanical and Electrical Engineering, Beijing University of Chemical Technology, Beijing 100029, China; 2021400188@buct.edu.cn; 2 College of Chemical Engineering, Beijing University of Chemical Technology, Beijing 100029, China; 2019200129@buct.edu.cn (Y.Z.); 2019210101@buct.edu.cn (F.Z.)

**Keywords:** high-pressure heat exchanger for hydrogenation unit, inferior crude oil, ammonium salt crystallization, numerical simulation, corrosion

## Abstract

The corrosion failure of the high-pressure heat exchanger in a petrochemical enterprise was simulated. A multiphase flow and heat and mass transfer simulation shows that the vortex core with a higher gas phase content and lower temperature is the region of easy crystallization, located on both sides of the center of the tube. The crystallization process occurs in the gas phase. As the reaction progresses, the crystallization range spreads from the tube wall to the center of the tube bundle, and the inner diameter of the tube bundle decreases continuously. In Fluent, a user-defined function, based on the ammonium chloride crystallization reaction, is loaded. The results show that crystallization first occurs in the tube bundles on both sides of the center of the tube and that the corrosion is aggravated by the erosion wall surface of crystal particles at the elbow, which is consistent with the actual corrosion failure location.

## 1. Introduction

With the aggravation of heavy and inferior crude oil, the content of chlorine, sulfur and nitrogen in inferior crude oil is increasing day by day. In the petrochemical production process, hydrogenation products of chlorine, nitrogen and sulfur are ubiquitous in the hydrogenation reaction effluent system due to its production characteristics. These impurities lead to the corrosion and leakage of operating equipment in the hydrogenation unit. Among them, the main corrosion of the high-pressure heat exchanger in the cooling equipment is multi-phase flow erosion and ammonium salt corrosion because of its high-pressure working conditions and the particularity of the multi-phase medium containing oil, water and gas [1]. Ammonium salt corrosion is an important reason for equipment failure in the heat exchange system of the hydrogenation unit. When the process is cooled, solid NH_4_Cl salt is formed by gaseous NH_3_ and HCl and deposited on the inner surface of the pipeline or equipment. When the deposited salt is diluted in water, a high concentration of the NH_4_Cl solution may be produced. NH_4_Cl is highly corrosive when it is close to the dew point of water or when the concentration is high, which will lead to the corrosion failure of the heat exchanger, affect the normal operation of equipment and bring huge economic losses [2]. Therefore, the safety of the heat exchanger has become an important hidden danger in the chemical industry and the corrosion of ammonium salt has also attracted extensive attention of researchers.

In order to prevent, reduce or even avoid safety accidents caused by ammonium chloride corrosion, a large number of studies on the reasons and corrosion properties of ammonium salt crystallization have been carried out. They are mainly divided into mechanism studies, experimental studies and numerical simulation studies of ammonium salt crystallization. Various methods are used to explore the corrosion mechanism. Ossai et al. summarized the causes of pipeline corrosion, including physical, chemical, environmental and material properties, and pointed out the consequences and damage degree of pipeline failure [3]. Liu et al. [4] used thermodynamic calculation to fit the crystallization curve and the actual working curve to determine the crystallization temperature of NH_4_Cl. Based on that, Zhu et al. [5] explored the influence of factors such as the raw material content and pressure on the crystallization temperature, obtained an empirical formula of the crystallization rate of ammonium salt and preliminarily studied the trend of the dynamic process [6]. Some scholars also carried out experimental studies on the corrosion behavior of the ammonium chloride solution, obtained corrosion-resistant materials through experiments and obtained the corrosion change trend based on environmental factors affecting corrosion [2,7,8,9,10,11,12]. However, there is much research on the corrosion of ammonium salt in an air cooler, but little research on the corrosion of ammonium salt in a heat exchanger. Ou et al. [13] believed that the interaction between the multiphase flow and air cooler structure leads to the corrosion of the tube bundle and affects the crystallization position. CFD analysis shows that the location of the maximum shear stress is subject to erosion risk. Through a simulation analysis of the temperature and distribution of ammonium chloride, Zhu et al. believed that an insufficient water injection caused corrosion under the ammonium salt scale [5]. Zheng et al. [14] simulated the position of the ammonium chloride deposition and the corresponding position of maximum concentration. The reason for the ammonium salt in the air cooler was analyzed from multi-flow field coupling, and the corrosion rate under scale was measured through experiments that were consistent with the actual situation and well verified [15]. Jin et al. [16] believed that the difference in the distribution of the multiphase flow field was the cause of corrosion thinning. The pipeline area with a high liquid content and high particle mass flow rate had a high risk of local corrosion thinning, and there was acid corrosion caused by ammonium chloride deliquescent. However, most of the current research on the corrosion cause only focuses on the distribution of multiphase flow field, whereas there are less analyses of the distribution of each phase and the mass transfer of the crystal phase in the heat exchange equipment. In addition, the specific behavior of the crystallization corrosion of the multiphase flow is rarely studied, and needs to be supplemented and developed continuously.

The aim of this paper is the analysis of the multiphase flow and heat and mass transfer for ammonium chloride crystallization in the high-pressure heat exchanger of a hydrogenation unit. During the operation of the heat exchanger, tube blockage and leakage appears. The crystallization of ammonium salt and corrosion failure mechanism of the heat exchanger tube bundle in the hydrogenation unit were studied, and the failure equipment was analyzed by numerical simulation. The multi-phase flow field and temperature field were simulated by establishing a model of the heat exchanger and using C language to develop the ammonium salt crystallization model for Fluent three-dimensional multi-phase flow calculation. The multi-phase flow and heat and mass transfer process determined the position of the corrosion of ammonium salt, resulting in the crystallization difference between different tube bundles. The results show that the ammonium salt is first generated in the gas phase, mainly distributed around the tube wall and gradually gets closer to the center of the tube bundle along the tube wall. The region with a higher gas phase content and lower temperature is the position of the easily crystallized tube bundle, which is located at the top corner of the first tube side of the heat exchanger and the bottom corner of the corresponding second tube side. In addition, the crystalline particles erode the outer bundle at the elbow and aggravate the corrosion. At the same time, scale corrosion and acid corrosion occur, caused by ammonium salt deliquescence in the area of easy crystallization.

## 2. Overview of Corrosion Failure

### 2.1. Technological Process

In this paper, the corrosion system in petrochemical enterprises is mainly based on direct diesel, coking gasoline and diesel and a small amount of catalytic diesel, such as raw material IV diesel and national V diesel. The processing capacity is 375 t/h, and the contents of sulfur, chlorine and nitrogen in the raw material oil are 2441.5 ppm, 0.92 ppm and 337.4 ppm, respectively. Five high pressure heat exchangers are designed. The reaction products first pass through two reaction products/mixed hydrogen oil heat exchanger (E-101A/B), then through the reaction products/low oil heat exchanger (E-102) and finally through the reaction products/cold mixed hydrogen oil heat exchanger (E-104A/B) to the reaction product heat exchanger (EA-101). In the cooling process of the reaction effluents, the corrosive components, such as ammonia, hydrogen chloride and hydrogen sulfide, that are formed after the hydrogenation reaction form crystals and may deposit on the surface of the tube bundle. Under certain conditions, this will lead to local deposition corrosion. The reaction product/cold mixed hydrogen oil heat exchanger E-104A is the corrosion failure equipment of this study. The heat exchanger has a double-shell tube structure, the tube diameter of the bundle is 19 mm, the one-way tube length is 7 m, the material is 15CrMo, the operating pressure is 7.78 MPa and the inlet temperature is 185 °C. The inlet temperature of the shell side is 129.8 °C, the outlet pressure is 8 MPa and the material is 15CrMoR.

### 2.2. Failure Case

The high-pressure heat exchanger E104 is a typical horizontal double pass U-shaped tube and shell heat exchanger. The inlet and outlet of the heat exchanger are vertically arranged, in which the inlet of the pipe pass is in the upper of the heat exchanger and the outlet is in the lower; the inlet of the shell side is in the lower of the heat exchanger and the outlet is in the upper. In 2016, when the factory maintained the heat exchange equipment, it was found that ammonium chloride scaling was serious at the outlet of the E-104A pipeline and that there was leakage at the elbow due to perforation.

**Figure 1:**
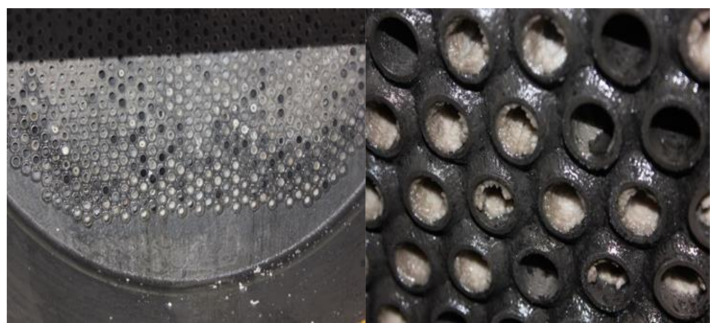
Scaling morphology of the heat exchanger tube bundle.

**Figure 2:**
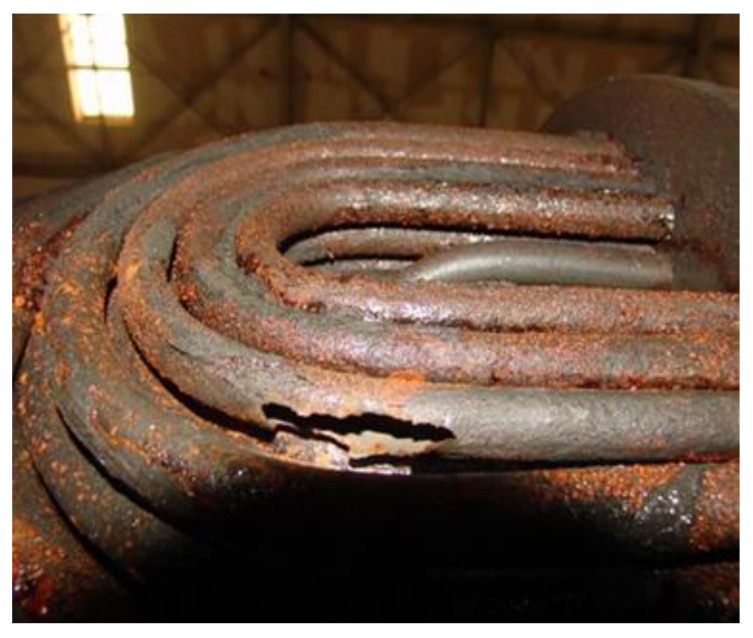
Perforation morphology of the heat exchanger tube bundle.

The maintenance personnel of the factory provided the corrosion failure morphology of the equipment, mainly for the scaling and perforation of the heat exchanger tube bundle, as shown in Figure 1 and Figure 2. It can be found that the tube bundle at the bottom corner of the outlet of the heat exchanger contains more white crystals, and that more white crystals are accumulated at the tube wall, resulting in a reduced circulation area and tube blockage. The perforation at the bend of the u-shaped tube may be caused by the multi-phase flow erosion of the medium in the tube during the flow of fluid. Tang [17,18,19] et al. believed that the multi-phase flow would undergo electrochemical corrosion and scouring corrosion during the flow and that the phenomenon of flow acceleration corrosion would occur. A chemical analysis was carried out on white crystal samples in order to obtain the element content of corrosion products, as shown in Table 1. The results showed that the chlorine content was high, which was presumed to be ammonium chloride crystal. The corrosion of ammonium salt may also occur during the operation of the heat exchanger.

## 3. Analysis of the Crystallization Process

### 3.1. Determination of Crystallization Temperature

**Figure 3:**
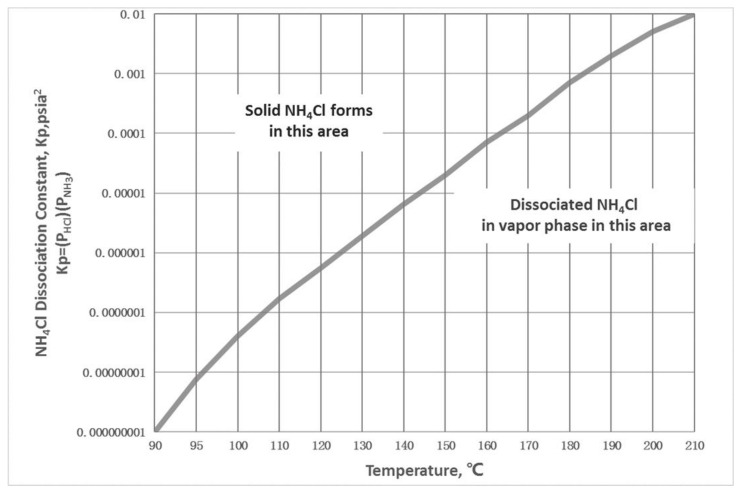
Measured data of ammonium chloride crystallization industry.

The crystallization data of ammonium chloride were collected by literature, and the original data were summarized to obtain the crystallization curve of ammonium chloride [20,21], as shown in Figure 3. NH_4_Cl crystallization was generated according to Equation (1).

NH_4_ (g) + HCl (g) → NH_4_Cl (s) (1)

After the fitting, the relation diagram of the t-lgKp ammonium chloride crystallization curve is obtained and the corresponding relation is Equations (2) and (3):

lg (Kp) = −0.0001186T^2^ + 0.0880699T − 13.5762237 (2)

T = 0.0769 (lg (Kp))^3^ + 1.6404(lg (Kp))^2^ + 218.45 (3)

Kp = P_NH3_·P_HCl_ (4)

**Figure 4:**
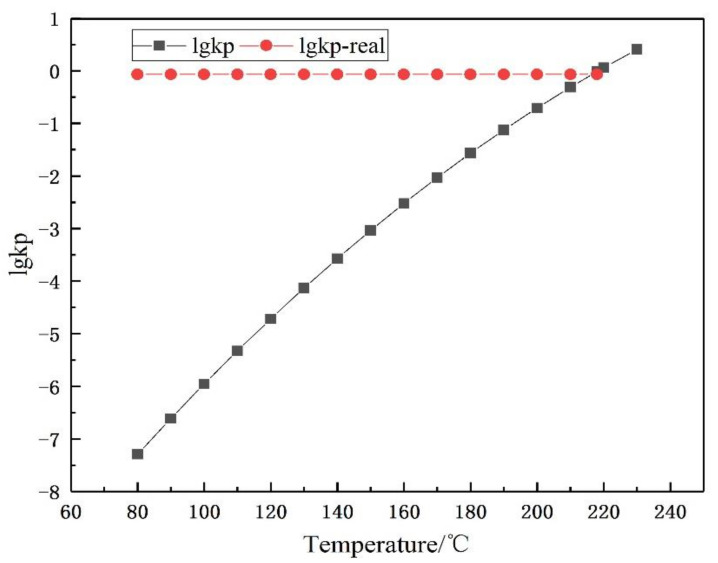
Determination of crystallization temperature of NH_4_Cl.

According to the operating conditions provided by the factory, the actual operating line is obtained by fitting. The crystallization curve of ammonium chloride intersects the operation line and the crystallization temperature is determined to be 216.5 °C, as shown in Figure 4. It can be known that the common part of the two curves is the crystallization region. The critical condition of crystallization is lower than the crystallization temperature, or the equilibrium constant is greater than the corresponding value in the crystallization curve. The calculation of Kp is shown in Equation (4). According to the process conditions, the inlet temperature of the heat exchanger in this paper is 185 °C, so there is a risk of crystallization in the whole heat exchanger.

**Figure 5:**
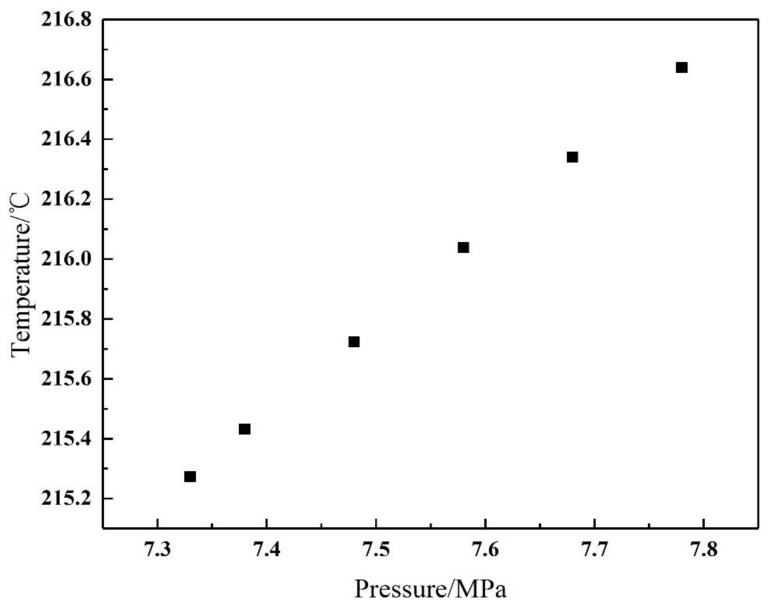
Effect of pressure on crystallization temperature.

**Figure 6:**
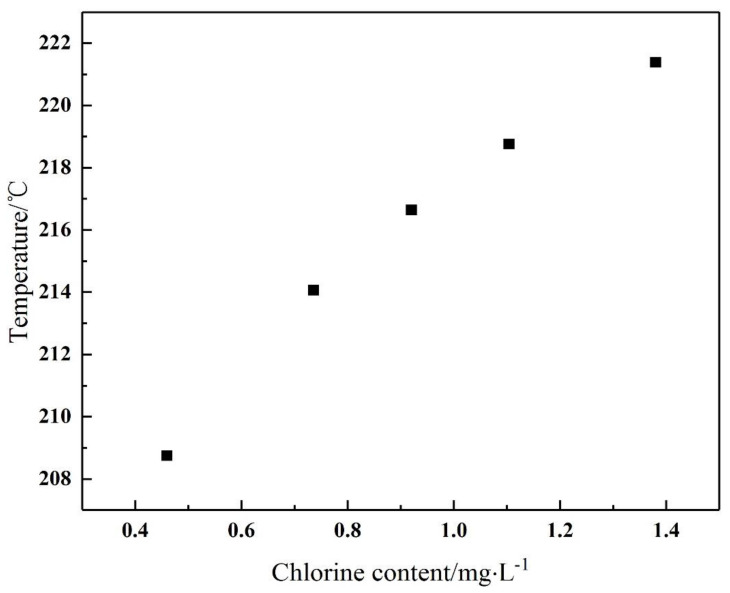
Effect of chlorine content on crystallization temperature.

In order to study the influence of the pressure, raw material content and other factors on the crystallization temperature of ammonium chloride, the crystallization temperature can be obtained by making operation lines under different conditions and intersecting the crystallization curve. Figure 5 shows the distribution of the crystallization temperature at different pressures. It can be seen that the corresponding temperature change within the range of the pressure drop is approximately 1 °C, so the influence of pressure on the crystallization temperature can be ignored. The different chlorine content of the raw material has a relatively great influence on the crystallization temperature. The higher the chlorine content of the raw material, the greater the mole fraction of hydrogen chloride in the gas phase, and the corresponding crystallization temperature will also become higher, as shown in Figure 6. Therefore, the crystallization temperature should be determined according to the actual operating conditions, and the production amount of ammonium chloride is different at different temperatures.

### 3.2. Crystallization Mechanism of Ammonium Salt

**Figure 7:**
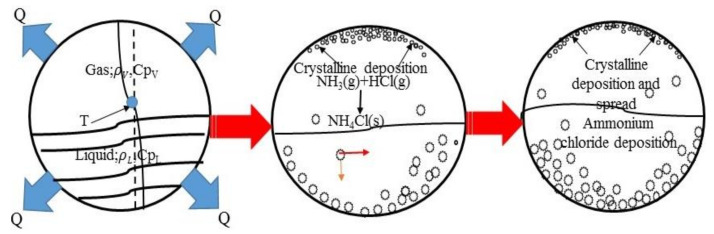
Crystallization and deposition corrosion mechanism of ammonium salt.

After the reaction, the medium flows into the heat exchanger and begins to carry out heat transfer between fluids. As long as there is a temperature difference, heat transfer will occur, so the fluid and metal in the bundle also have heat transfer. After the fluid flows out of the reactor, the temperature is high. Ammonia gas and hydrogen chloride are in a gaseous phase. Due to the small specific heat of the metal in the tube wall, there is a large temperature difference in the tube wall under the condition of absorbing the same heat. When the critical reaction condition is reached, the generated ammonium salt will first attach to the upper tube wall of the tube bundle and accumulate continuously around the tube wall. The generated white crystals will also flow or dissolve with the fluid. When a large number of ammonium salt particles are generated and accumulated, the phenomenon of tube blockage will occur. In this process, under the influence of gravity, white crystals will fall to the lower part of the bundle, and continuous accumulation may lead to the phenomenon of ammonium salt deposition. To sum up, ammonium chloride crystallization in the bundle occurs according to this mechanism. Each bundle is affected by uneven flow field distribution, flow condition and temperature distribution, and the final amount of ammonium salt generated will be different. The formation process and corrosion mechanism of the crystallization and deposition of ammonium salt are shown in Figure 7, which can explain that the white crystals in the tube bundle in the figure are clustered around the tube wall in the field failure.

By analyzing the mechanism diagram of the crystallization and deposition corrosion of ammonium salt, it can be seen that the multiphase flow field, temperature field and component distribution determine the multiphase flow and heat and mass transfer, affect the changing trend of flow field and temperature component distribution and lead to the difference in crystal distribution between the tube bundles in the heat exchanger. Therefore, by analyzing the fluid flow and heat and mass transfer phenomenon in the heat exchanger, the easy crystallization area and the serious corrosion position can be determined.

**Figure 8:**
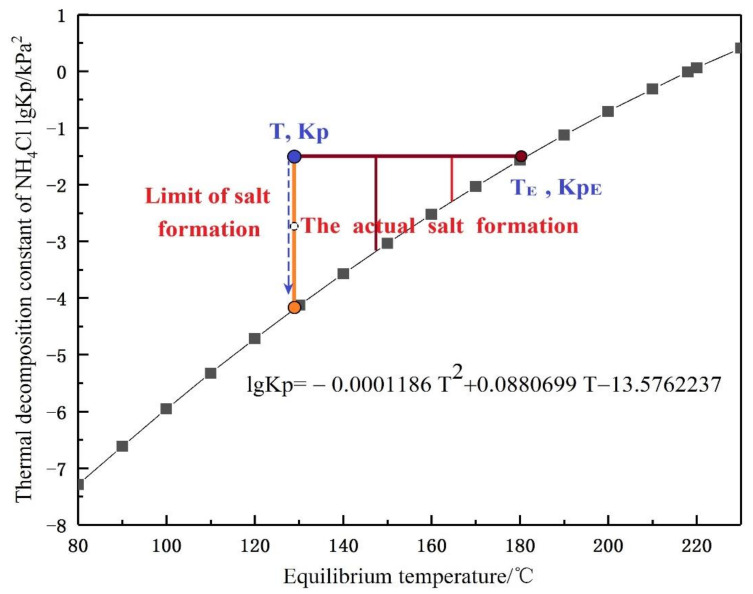
Crystal equilibrium curve of ammonium chloride.

The phase diagram of ammonium salt crystallization mainly shows the corresponding relationship between the temperature and actual lgKp, as shown in Figure 8. Salt formation varies at different temperatures. A low temperature is conducive to the occurrence of ammonium salt crystallization. Based on this phase diagram, the actual situation of salt formation in the heat exchanger can be divided into three kinds: crystallization reaction occurs; crystallization reaction reaches equilibrium; crystallization reaction does not occur. The actual limit of the salt formation depends on inlet conditions, such as low content components and the operating temperature.

### 3.3. User-Defined Function Calculation Principle

In order to study the distribution of ammonium salt in the heat exchanger, combined with the specific analysis of the crystallization process, this paper mainly programmed the calculation model of the crystallization reaction HCl + NH_3_ → NH_4_Cl and obtained the s source term user-defined function that defines the actual crystallization rate of ammonium salt, which is used for the flow heat and mass transfer of three-dimensional multiphase flow. The generation of ammonium chloride was obtained by simulation calculation. The preparation of the source term user-defined function is mainly based on the crystallization curve as the basis to judge whether ammonium chloride generation occurs. When the temperature difference limit is small, the reduction in hydrogen chloride or ammonia gas is considered as the generation of ammonium chloride. On this basis, the formula of the crystallization rate is obtained as Equation (5).

Speed = (P_HCl_ − P_HCl-t_)/Pres/δt × n × M_NH4Cl_ (5)

where P_HCl_ is the actual partial pressure of hydrogen chloride; P_HCl-t_ is the partial pressure of hydrogen chloride on the corresponding crystallization curve at the operating temperature, namely the equilibrium partial pressure; n is the number of moles; M_NH4Cl_ is the molecular weight of ammonium chloride; δt is a time step and can be set in the process of numerical simulation. Pres is the actual total pressure.

**Figure 9:**
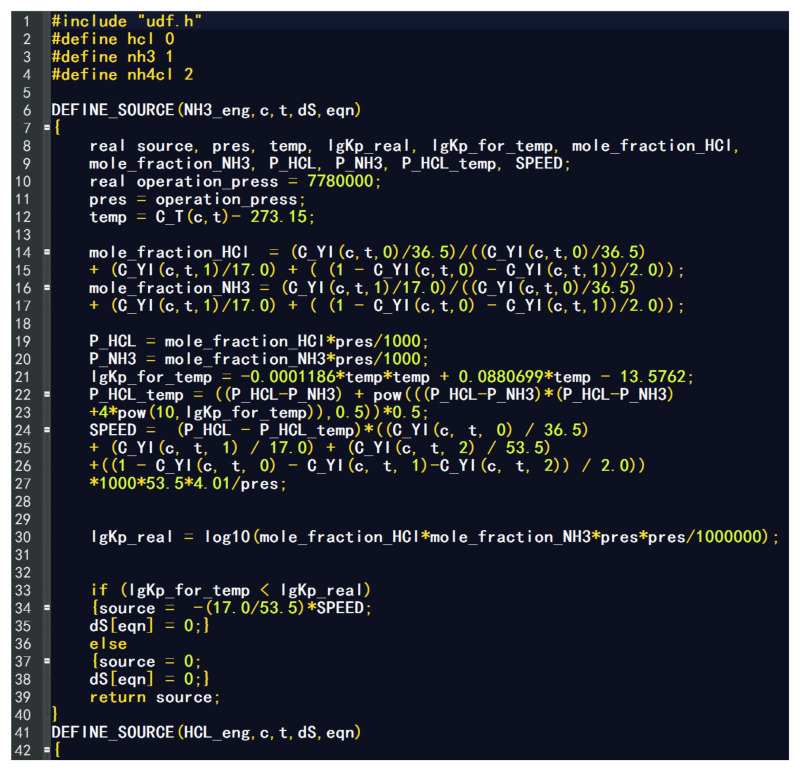
The core code of user-defined function.

The compiled source term user-defined function core code is shown in Figure 9:

During the calculation process, the crystallization reaction can be determined by determining the relationship between the temperature at the entrance and the product of gas phase partial pressure. By comparing the amount of Kp_real and lg (Kp) in Equations (2) and (4), there are three cases:

1. Kp_real > lg (Kp). At this point, the inlet phase conditions meet the requirements of the ammonium salt crystallization reaction and gradually tend to balance with the progress of the reaction. The result is that ammonium salts are produced;
2. Kp_real = lg (Kp). At this time, the inlet phase condition just meets the critical requirements of the crystallization reaction of ammonium salt and will be in the crystallization equilibrium state;
3. Kp_real < lg (Kp). At this time, the inlet phase condition does not meet the temperature or material conditions for the crystallization reaction of ammonium salt; that is, no ammonium salt is determined.

Therefore, the crystallization reaction can be determined by judging the temperature and gas partial pressure product of any tube bundle at any time in the calculation process. If the crystallization reaction can be carried out, the corresponding crystallization rate is given and the reaction can be carried out under the given conditions. The generation of ammonium salt is a dynamic process, so, in the process of iterative calculation, the remaining gas phase at the last moment is regarded as the reference value at the next moment, which is the same as the determination method of the inlet, and the cycle repeats until the calculation converges. Convergence is judged based on the mass, momentum and energy transport conservation for the whole field and each control unit. During each iteration, user-defined functions are called for simulation calculation.

### 3.4. Numerical Methods and Modeling

**Figure 10:**
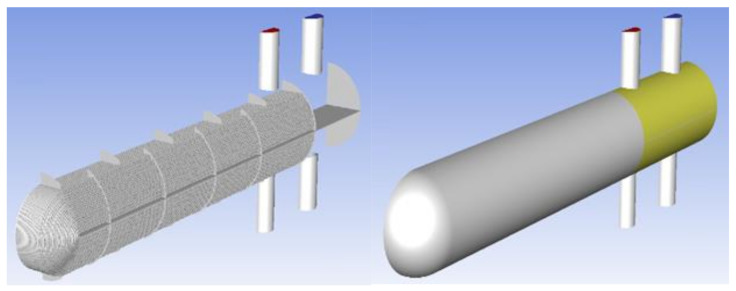
Schematic diagram of heat exchanger model.

Through the actual investigation of field working conditions, relevant data were obtained to establish the heat exchanger model, as shown in Figure 10.

According to the symmetry characteristics of the 3D geometry model, a 1/2 geometry structure is selected as the computational domain model. Based on the two-dimensional grid, the three-dimensional grid model is gradually established by stretching, rotation, translation, mirroring, merging and other operations. The number of grids is approximately 100 million. In Fluent, the mixture model is set to calculate the flow of gas and oil phases, the K-ω model of SST is used to solve the temperature field and flow field and the non-slip wall condition is used to calculate. In the setting of the boundary conditions, the inlet temperature of the pipe side is 185 °C, the inlet temperature of the shell side is 129.8 °C, the inlet type is a mass flow inlet and the outlet is a pressure outlet. The governing equations adopted in this paper include the law of conservation of mass, the law of conservation of momentum and the energy conservation equation. The corresponding boundary conditions are shown in Table 2 and Table 3. After initialization, a relevant simulation calculation is initiated.

## 4. Results and Discussion

### 4.1. Multiphase Flow Field Analysis

**Figure 11:**
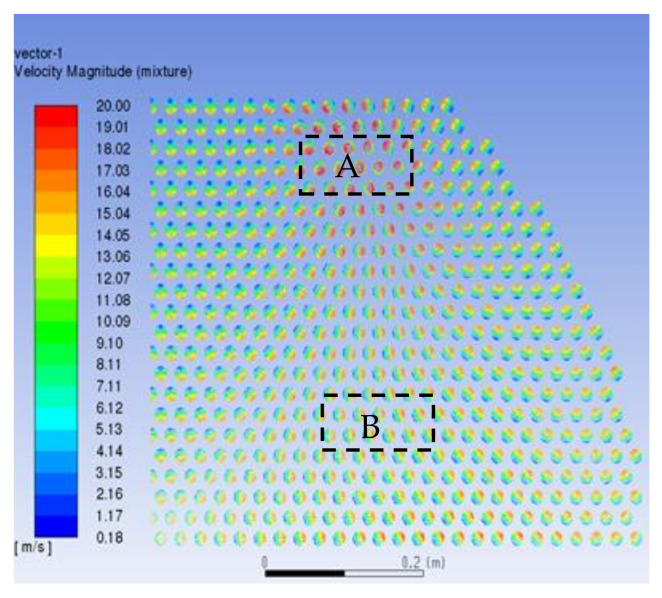
Velocity vector diagram of tube bundle inlet. (**A**) the vortex core region and (**B**) the peripheral area.

**Figure 12:**
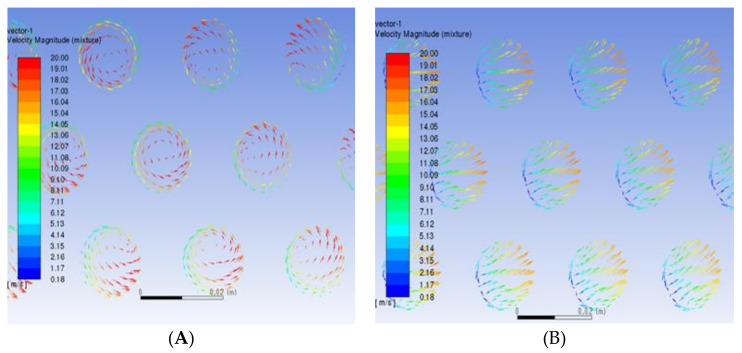
Local velocity vector diagram of tube bundle inlet: (**A**) the vortex core region and (**B**) the peripheral area.

When the fluid enters the heat exchanger, it will produce backflow and influence the direction. The fluid working medium will sweep the end face of the tube mouth horizontally. If the crystal particles have been produced by the flow, impact corrosion will be caused. The velocity distribution of tube bundles corresponds to the inlet, as shown in Figure 11. The maximum velocity is 20 m/s. The mass flow rate into each pipe will remain the same after the inlet, so the pipes with a large mass flow will maintain a higher velocity and the velocity distribution in each pipe is slightly different from that in the inlet; that is, the velocity in the upper half of the pipe is high, whereas the velocity in the lower half is low. Figure 12 shows the partially enlarged velocity vector diagram of the pipe inlet. (A) is the tube bundle at the vortex center of the reflux area and the fluid rotates into the heat exchange tube in a small vortex; (B) is the velocity vector diagram of the tube bundle in the peripheral area. It sweeps through the inlet in the direction of the tangential velocity of the vortex and enters the heat exchange tube in the direction of the velocity component along the pipe. According to the symmetry of the heat exchanger, there are two corresponding vortex core regions.

**Figure 13:**
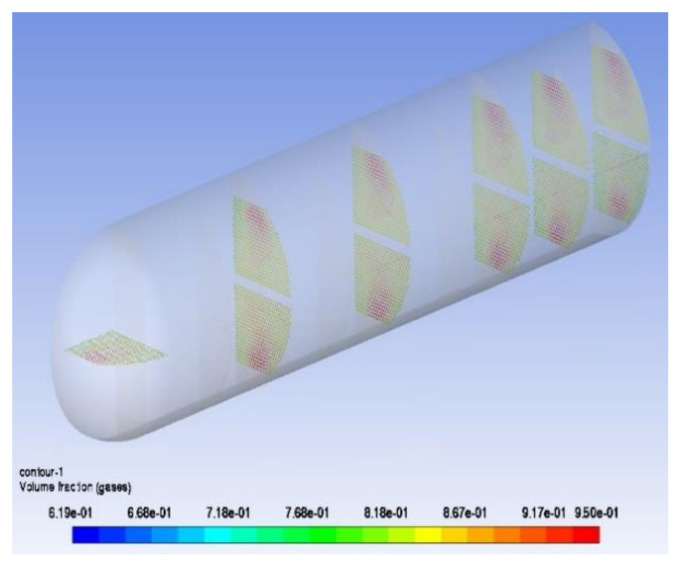
Distribution law of gas volume fraction along pipe.

**Figure 14:**
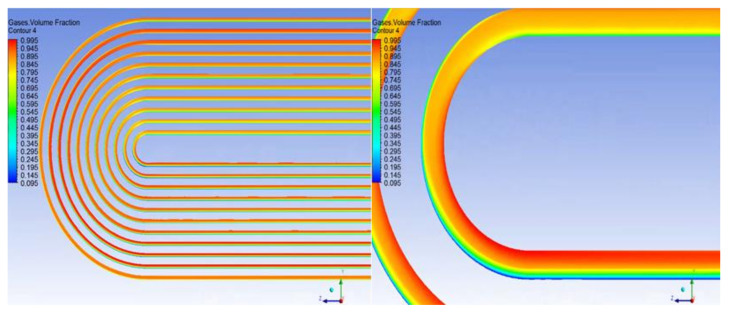
Gas volume fraction distribution in bend area of heat exchange.

**Figure 15:**
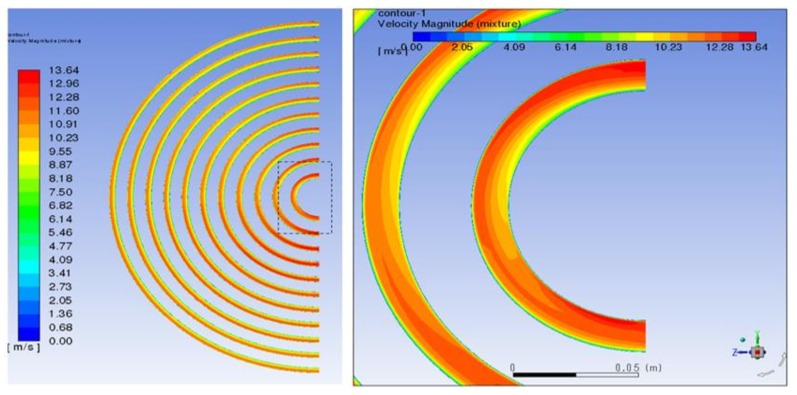
Velocity distribution in the bend area of heat exchange.

**Figure 16:**
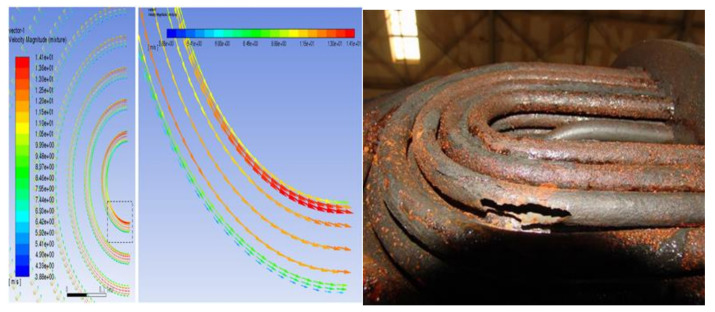
Comparison between simulated results of the elbow area and actual perforated area.

As a result of the centrifugal force of the swirling flow, the liquid diesel with high density moves to the outside of the vortex, whereas the gas with low density tends to distribute in the inside of the vortex, and the working medium component proportion forms an uneven distribution. Figure 13 shows the gas phase distribution along different sections of the pipe. The composition of different pipes is different and the gas content of the tube bundle area corresponding to the vortex core is larger, which is also an important reason for the different corrosion of each pipe. It can be seen from the gas phase volume fraction of the tube bundle that, at the end of the first tube, the gas phase and liquid phase flow through the U-shaped tube bend and form obvious stratification under the action of gravity and centrifugal force. It can be seen from Figure 14 that the liquid phase with high density is distributed on the outside of the tube bundle, whereas the gas phase is distributed on the inside of the U-shaped tube and continues to flow forward in the second tube side. As shown in Figure 15, the fluid flows through the elbow at a high velocity and may have crystallization particles, so solids are distributed on the outside of the elbow under the influence of centrifugal force in the flow process, move forward along the pipe with the fluid flow and are then distributed at the bottom of the second pipe under the action of gravity. At the same time, the crystal particles under the action of centrifugal force will also erode the outer wall of the tube bundle, resulting in elbow perforation and leakage. Figure 16 shows that the simulation result is consistent with the actual perforation position by comparing the velocity vector distribution at the elbow with the actual perforation position.

### 4.2. Heat Transfer Analysis

#### 4.2.1. Global Heat Transfer Analysis

**Figure 17:**
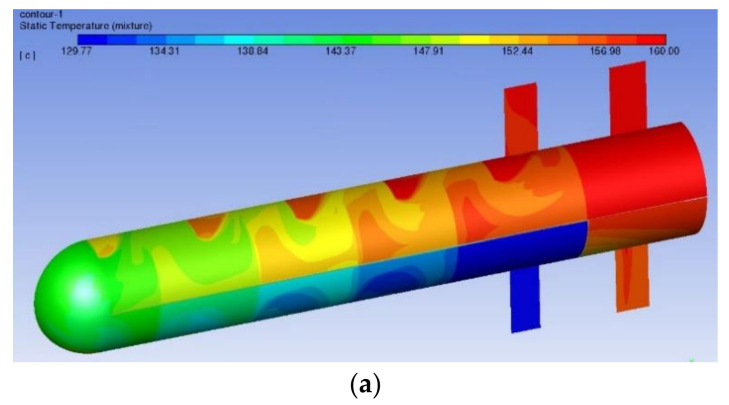

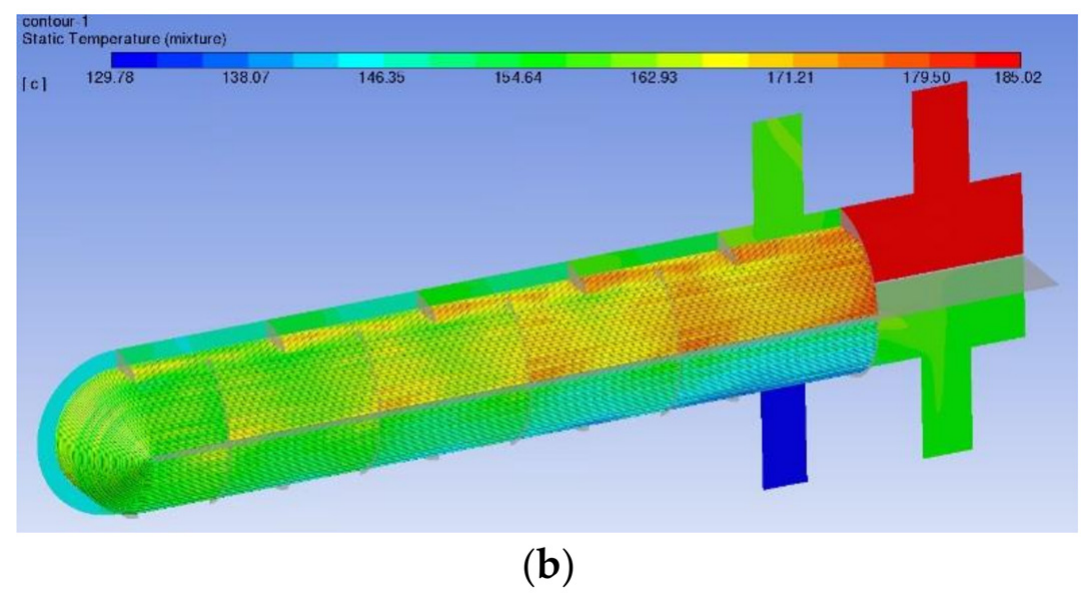
Temperature distribution of heat exchanger: (**a**) temperature of the tube side and (**b**) temperature of the shell side.

Figure 17 shows the temperature distribution of the shell side surface and the tube bundle wall of the heat exchanger. On the back side of the baffle plate, due to the existence of a reflux zone, the heat exchange effect is poor, resulting in a local high temperature. The temperature distribution of the heat exchange tube bundle is similar to the above. In the bundle section at the back of the baffle plate, the wall temperature is higher. As the heat transfer continues, the fluid temperature gradually decreases along the flow direction of the bundle.

**Figure 18:**
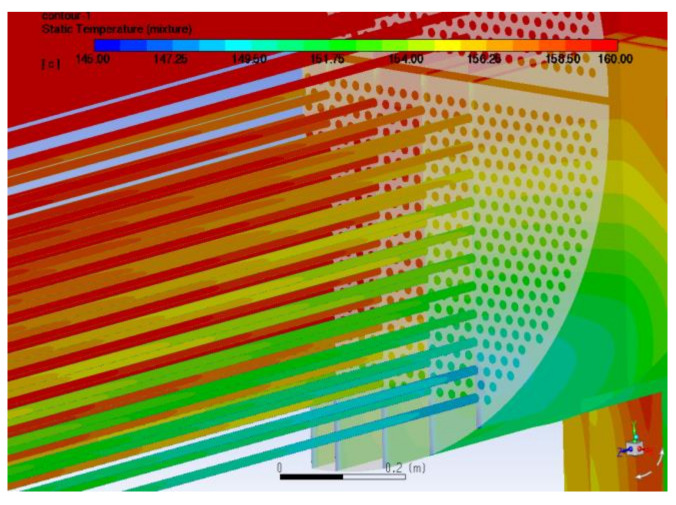
Temperature distribution in the outlet area.

Figure 18 shows the temperature distribution at the outlet of the tube side. After the tube process, the temperature at the outlet of different tubes already varies considerably, and the temperature difference in the mainstream area can reach approximately 10 °C. This temperature difference is also the reason for the serious salt build-up found in some areas during the actual maintenance.

#### 4.2.2. Local Heat Transfer Analysis of Tube Bundle

**Figure 19:**
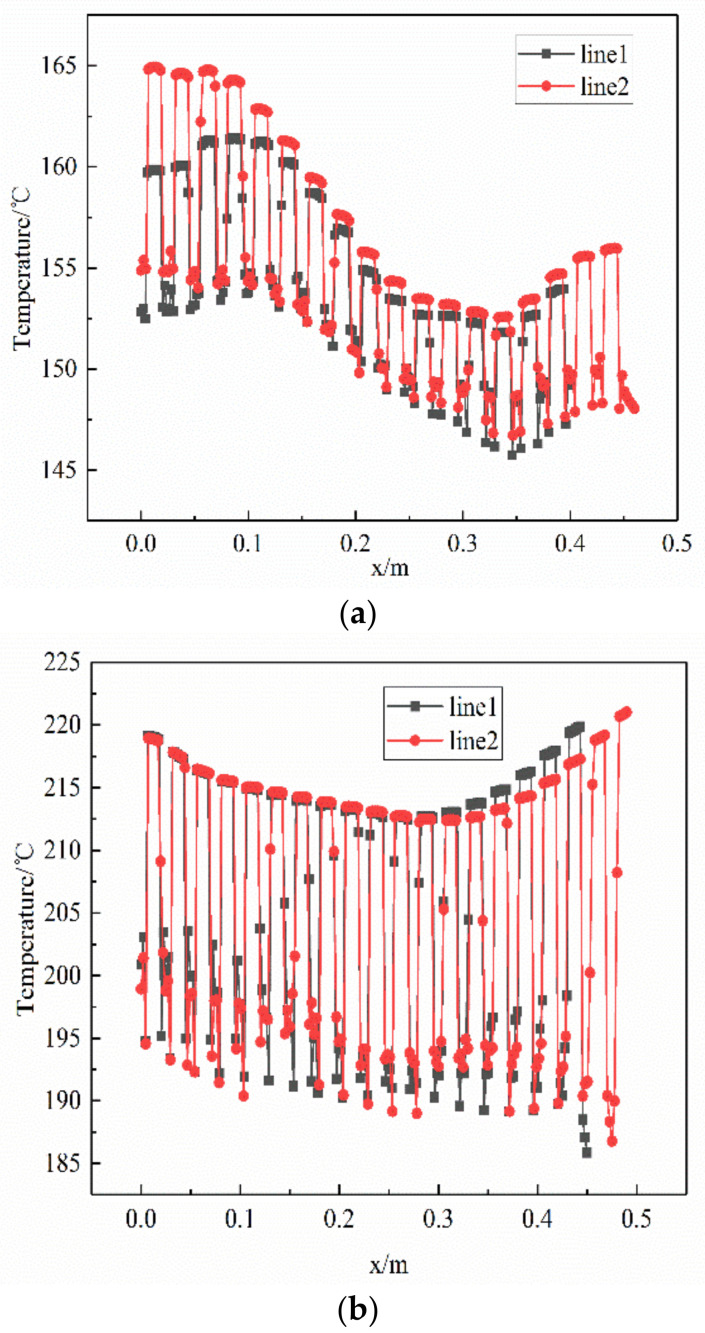
Wall fluid temperature distribution of the tube bundle: (**a**) the first tube side and (**b**) the second tube side.

The analysis of the velocity field of the multiphase flow shows that there is a backflow of the fluid and, therefore, that a vortex is formed. At the core of the vortex, the velocity is higher and the gas phase is more abundant. Two rows of tube bundles located in the vortex region at the outlet of the pipe side were selected for a local analysis of its temperature. Figure 19 shows the temperature variation of the tube bundle in the x-direction. The temperature of the tube bundle fluctuates along the x-axis and is lower between 0.1 m and 0.4 m. The temperature difference between the wall and the fluid in the tube bundle is approximately 10 °C, where the wall temperature is lower, and the energy change and transfer caused by the fluid flow may affect the temperature distribution of the whole system, resulting in a radial temperature gradient. The crystallization phase diagram shows that, under the same conditions, the tendency to crystallize is greater in the low temperature region and the amount of crystallization is higher at the low temperature position; hence, the crystallization process may start at the wall surface.

The physical parameters of the gas–liquid phase in the heat exchanger medium are shown in Table 4. Combined with the principle diagram of ammonium salt deposition, the specific heat capacity and mass product of the gas phase components can be ignored compared to the oil phase and water phase under the same heat absorption, so the temperature of the gas phase changes greatly. Under the same conditions, the temperature of the gas phase drops faster than that of the liquid phase, so it is easy for the gas phase environment to meet the critical conditions for the reaction, and ammonium chloride crystals are first produced in the gas phase, so the amount of the reaction depends on what is less abundant in ammonia and hydrogen chloride.

**Figure 20:**
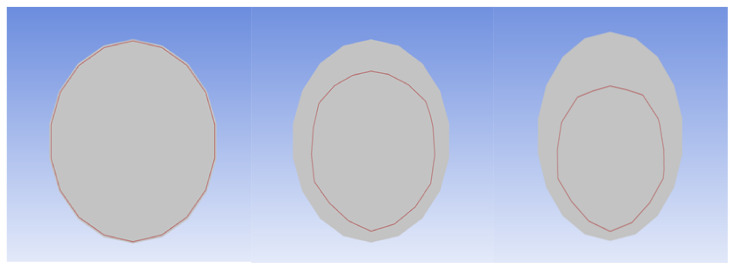
The change in isotherms along the flow direction.

The change in the 170 °C isotherm of the seventh tube bundle in the vortex core region along the flow direction is shown in Figure 20, which keeps approaching the center of the tube bundle along the tube wall, indicating that the low temperature region keeps increasing along the flow direction. The change trend of the low temperature area at the top of the tube bundle is faster than that at the bottom, which is consistent with the crystallization and deposition corrosion mechanism of ammonium salt. Therefore, with the progress of flow and heat transfer, the area of the low temperature salt formation in the tube bundle gradually increases and spreads from the tube wall to the center of the tube bundle and the inner diameter of the pipe becomes smaller, which leads to a decrease in the flow rate, increase in the pressure drop and finally leads to blockage and even a pipe burst. The heat transfer effect is mainly reflected in the different temperature distribution of the tube bundle, which affects the distribution of ammonium salt.

### 4.3. Mass Transfer Analysis

**Figure 21:**
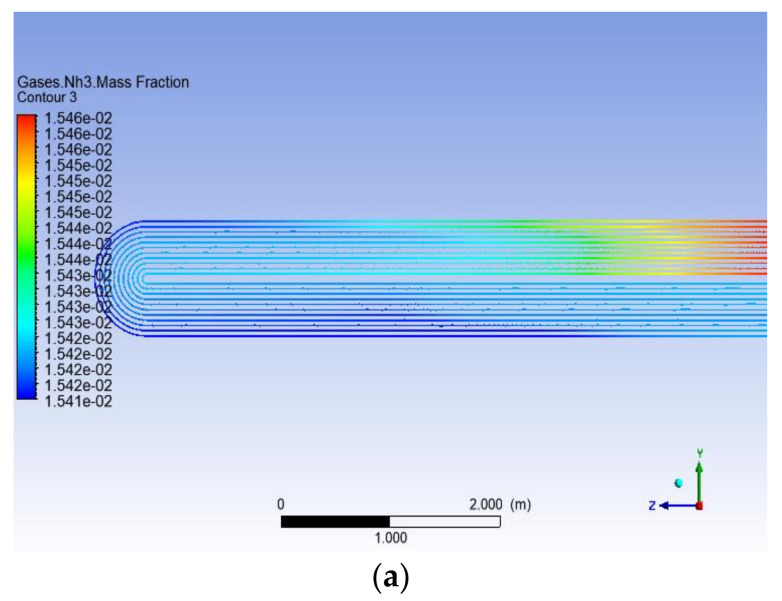

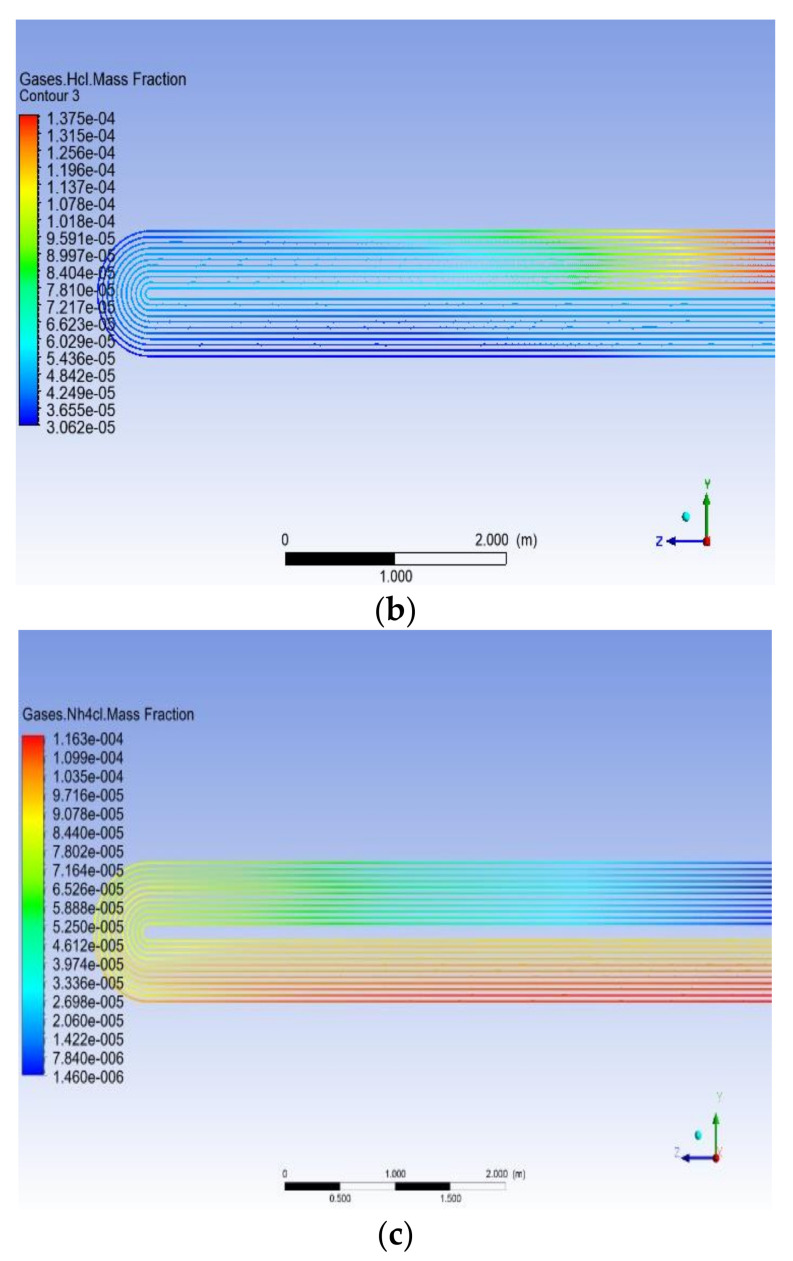
Changes in the content of the components: (**a**) ammonia, (**b**) hydrogen chloride and (**c**) ammonium chloride.

The heat exchange and chemical reactions in heat exchangers are based on heat and mass transfer processes, mainly performed according to the law of the conservation of mass, the law of the conservation of momentum and the equation of the conservation of energy, for three-dimensional compressible multicomponent fluids, including heat and mass transfer. After the fluid enters the heat exchanger, the energy equation comes into play. As the fluid flow and temperature change, the crystallization reaction begins when the corresponding critical conditions are reached and the corresponding reactant components change. Figure 21 shows the content changes in components NH_3_, HCl and NH_4_Cl. It can be seen that the content of the reaction components decreases gradually along the tube, whereas the content of products increases continuously, indicating that the combination of the user-defined function and Fluent is good.

**Figure 22:**
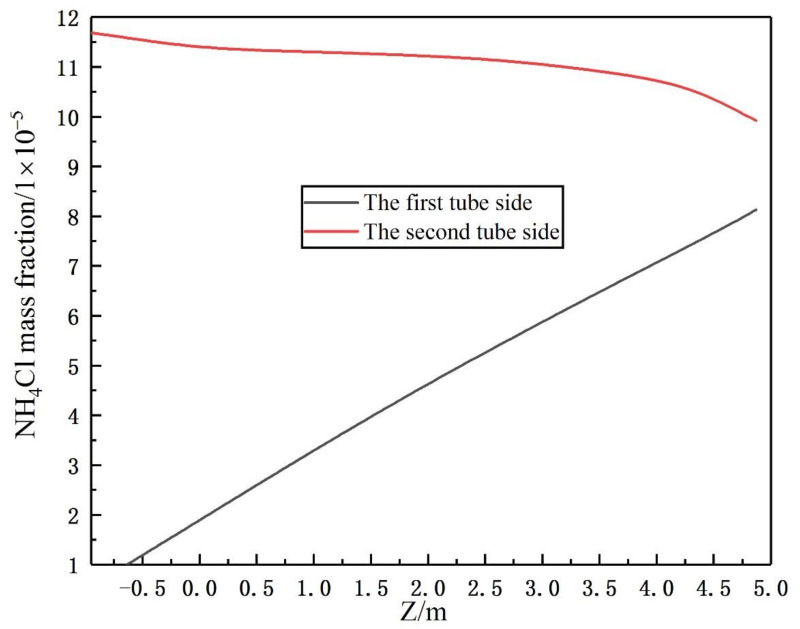
Crystal distribution of the seventh bundle.

The seventh bundle in the low-temperature region was selected as the research object. Figure 22 shows the crystal distribution near the tube wall. The heat exchange between two gas–liquid phases continues to strengthen along the flow direction, so the temperature keeps decreasing and the amount of crystallization keeps increasing. Crystals were found around the wall of the tube, indicating the formation of ammonium chloride in the gas phase. This is consistent with the rule in the crystallization phase diagram.

### 4.4. Determination of the Region of Easy Crystallization

The above analysis of the flow field and heat and mass transfer, combined with the distribution characteristics of the flow and gas phase concentration, shows that the temperature is lower at the vortex core due to the high gas velocity and high heat transfer capacity. In addition, because it is the region with a high gas concentration, the critical salt formation condition and generation of ammonium salt is easier to reach, and the corresponding bundle at the vortex core is determined to be the region with easy crystallization.

**Figure 23:**
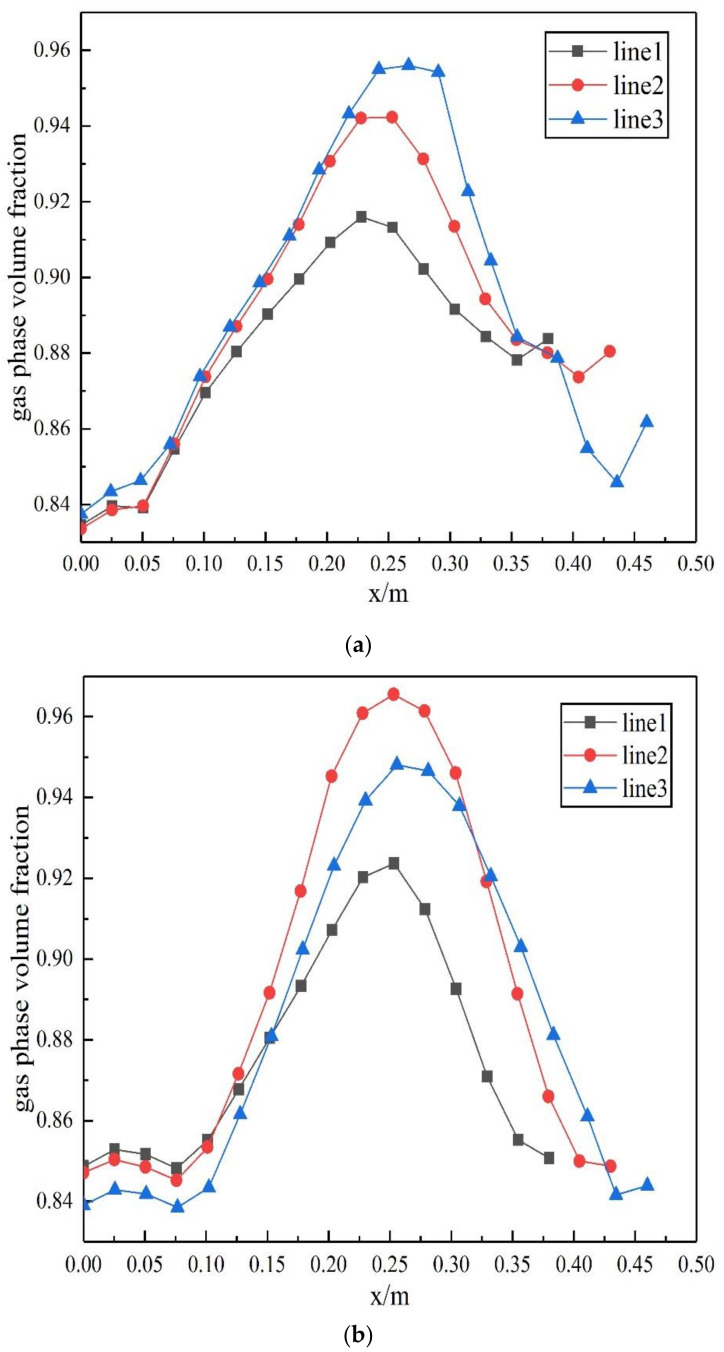

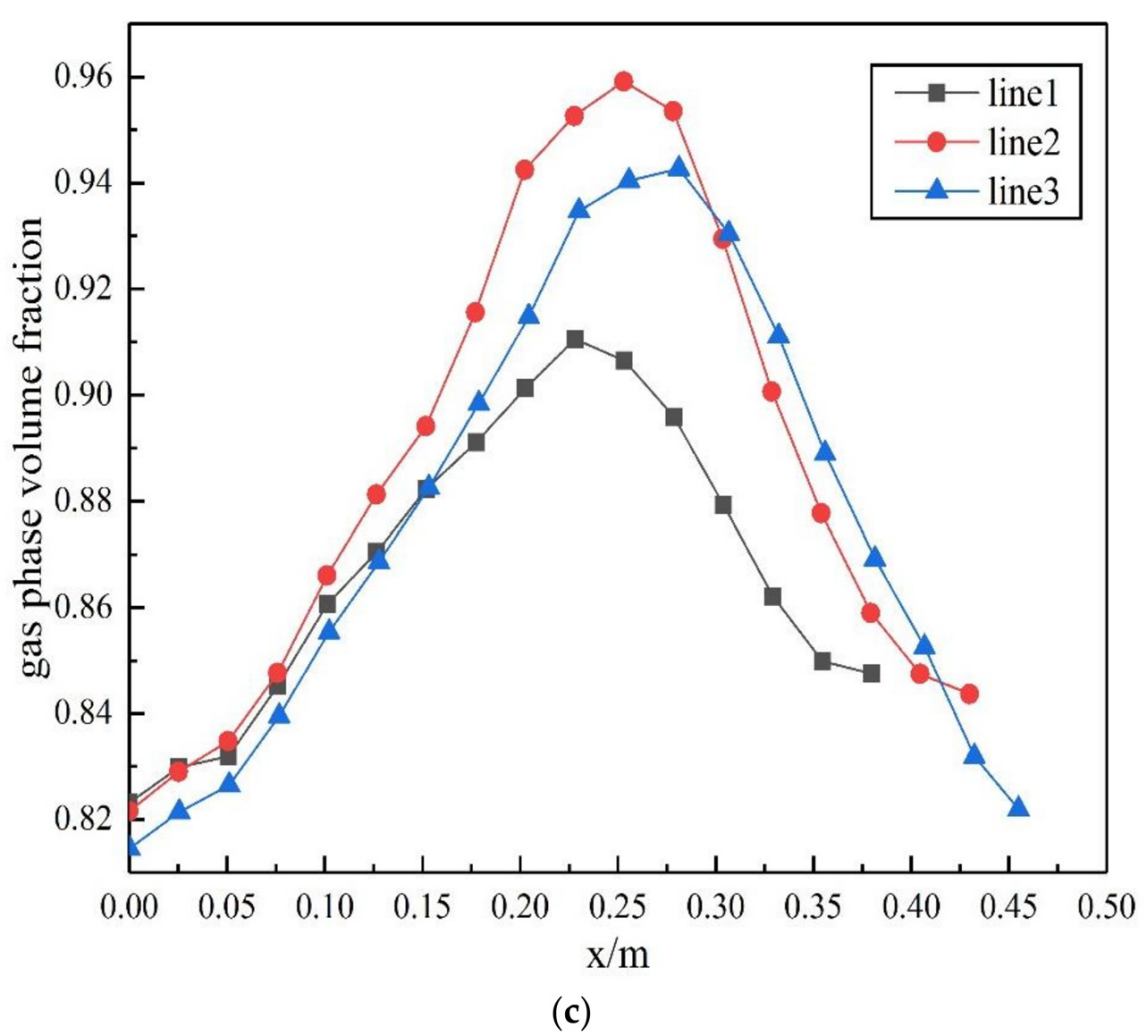
Distribution of the gas phase volume fraction in differentstages: (**a**) initial stage of calculation, (**b**) middle stage of calculation and (**c**) late stage of calculation.

**Figure 24:**
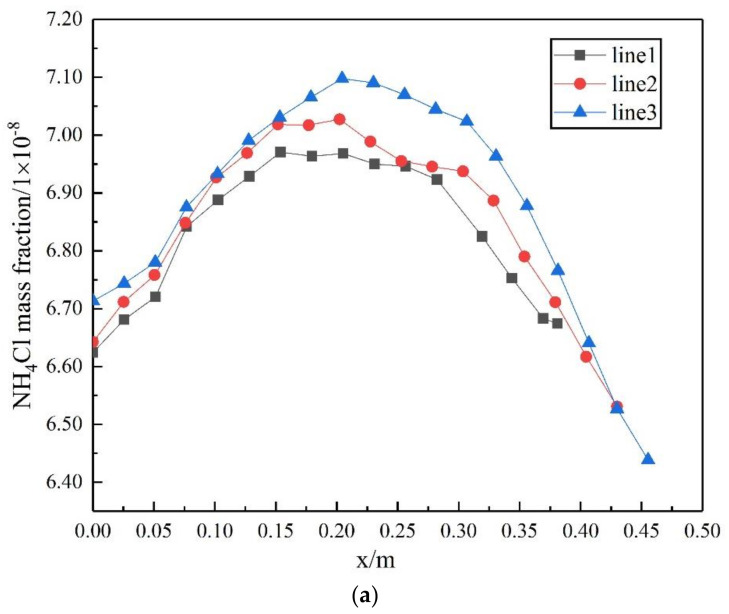

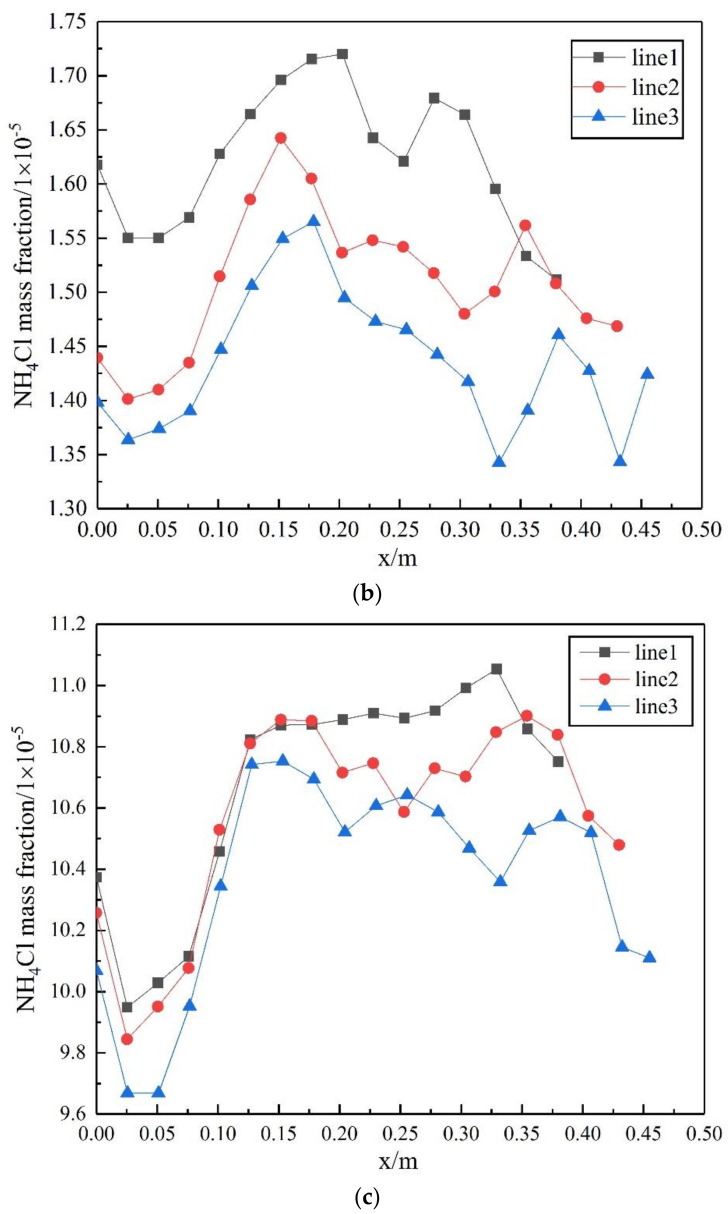
Distribution of the crystallization amount of ammonium chloride in tube bundles in different stages: (**a**) initial stage of calculation, (**b**) middle stage of calculation and (**c**) late stage of calculation.

The crystallization reaction proceeds to different degrees at different times and the heat exchange process is the interaction of fluids in a dynamic process of constant flow. In this study, three rows of tube bundles located in the area of easy crystallization, which belong to the X-Y section of the inlet and outlet of the heat exchanger, were selected to analyze changes in the gas phase content and crystallization amount at the initial, middle and late stage of calculation. Moreover, the region where ammonium chloride was first generated was identified. The gas phase distribution and ammonium salt content were obtained as shown in Figure 23 and Figure 24. The simulation results show that the gas phase content is the largest in the region of 0.15−0.35 m perpendicular to the direction of the fluid flow. At the beginning of the fluid entering the heat exchanger, the ammonium chloride crystallization reaction also begins. The vortex core region at the inlet of the tube side generates ammonium chloride at the earliest. As the reaction goes on, the amount of ammonium chloride keeps increasing and the maximum mass fraction of ammonium chloride in the vortex core region at the outlet of the tube side is 1.16 × 10^−4^.

**Figure 25:**
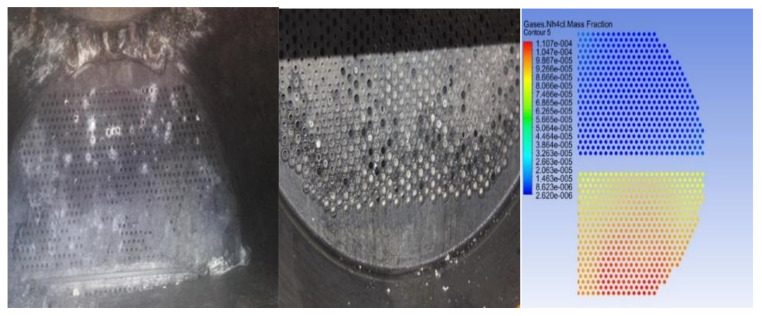
Comparison of actual corrosion locations to simulated results.

Figure 25 shows a comparison between the actual corrosion position and the distribution of ammonium salt in the inlet and outlet sections of the heat exchanger. The ammonium salt in the first pipe side is mainly distributed in the upper tube bundle and, after flowing, it is located at the bottom of the second pipe side, namely the vortex core region with the high gas phase content and low temperature, which is consistent with the actual location of ammonium salt crystallization. It shows that the simulation study in this paper has the value of guiding actual production, which can be used to predict the actual crystallization area of ammonium salt in the high-pressure heat exchanger of the hydrogenation unit and to reduce the loss of equipment and production caused by ammonium chloride corrosion.

### 4.5. Corrosion Risk of Ammonium Chloride

Reports indicate a higher risk of under-deposit corrosion in areas with s high ammonium content, and it is believed that the crystallization-prone areas in this study have corrosion failure caused by ammonium chloride crystallization in the following two main forms. One is under-deposit corrosion caused by ammonium chloride deposition. Crystallization particles are deposited on the wall of the pipe to form a closed area, which makes the difference between the media inside and outside the area. Therefore, a battery environment with a cathode and anode exists around the scale layer. The wall metal covered by the scale layer is the anode and the wall metal material around the scale layer is the cathode. Electrons flow between the cathode and anode faster than normal, resulting in electrochemical corrosion [22,23]. From the actual corrosion failure diagram, it can be seen that the scaling of the tube bundle and the existence of crystallization particles will also lead to the occurrence of multiphase flow erosion corrosion. The other is the acid corrosion caused by the deliquescence of ammonium chloride salt.

**Figure 26:**
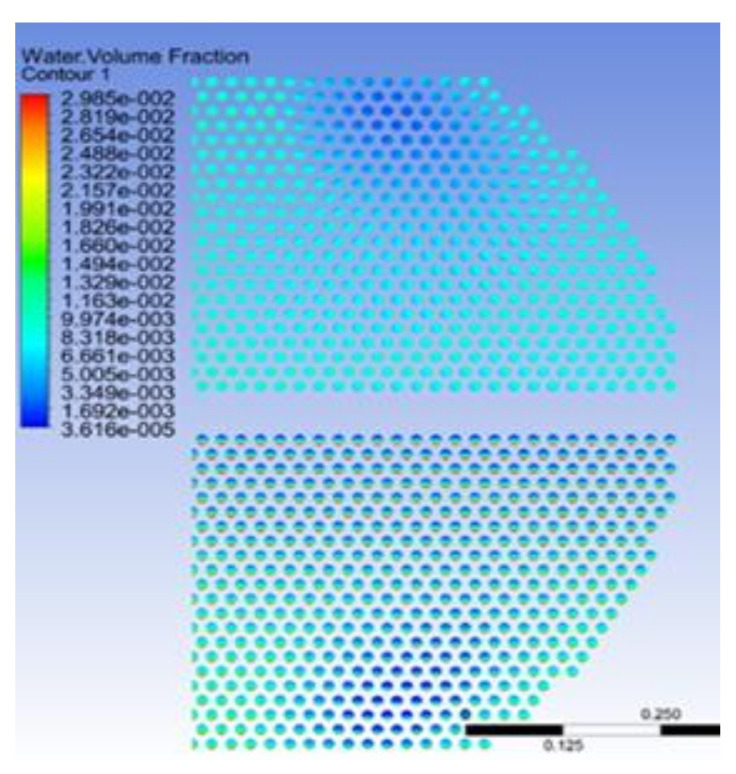
Distribution of the water phase.

As shown in Figure 26, the water phase distribution is uneven after the water injection, and is mainly distributed in the first pipe side and the bottom of the tube bundles in the second pipe side. The difference in the water phase distribution results in an uneven mixing of ammonium chloride and water. There is less water in the area prone to crystallization. When the deposited salt is diluted in water, a high concentration of the NH_4_Cl solution may be produced. The NH_4_Cl salt is highly corrosive when it is close to the dew point of water or at a high concentration level, so the risk of acid corrosion is higher in regions prone to crystallization.

## 5. Conclusions

The corrosion behavior of a high-pressure heat exchanger is studied in this paper. Based on the correlation technological process of the high-pressure heat exchanger in the hydrogenation unit, ammonium salt crystallization and the corrosion mechanism were studied and the inner flow field was simulated by using the mixture model. The conclusions of this work are given below:

1. The intersection of the actual operating line of the heat exchanger and the ammonium chloride crystallization curve determines that the crystallization temperature of ammonium chloride is 216.5 °C. The influence of pressure on the crystallization temperature is negligible, and the greater the chlorine content of the raw material, the higher the crystallization temperature becomes. In addition, there is a risk of crystallization in the entire heat exchanger;
2. According to the corrosion mechanism of ammonium salt crystallization, the specific heat capacity of the fluid is different from that of the metal wall during the heat exchange process, and there is a temperature difference. The specific heat capacity of the metal wall is small, so the temperature difference at the pipe wall is large and the ammonium salt will first deposit around the pipe wall. In the same way, the temperature drop of the gas phase in the multiphase flow component is the greatest, so the ammonium chloride crystallization reaction is generated in the gas phase. It is believed that the distribution of ammonium salt is determined by the flow of the multiphase fluid and the heat and mass transfer between the phases;
3. Based on the crystallization equilibrium phase diagram of ammonium chloride, the actual situation of salt formation in the heat exchanger can be divided into three kinds. Moreover, the calculation model of the crystallization reaction HCl + NH_3_ → NH_4_Cl was programmed to obtain the source term user-defined function, which is combined with Fluent software to simulate and analyze the heat exchanger;
4. The simulation results of the multiphase flow field show that the maximum velocity at the vortex core is 20 m/s and that the swirling flow causes more gas content here. The velocity at the elbow is large and the ammonium salt particles are distributed on the outside of the elbow by centrifugal force, which erodes the wall surface and causes corrosion more easily. The simulation results are consistent with the actual perforation location;
5. The heat and mass transfer simulation results show that the fluid temperature is relatively low at the vortex core. The low temperature area of the tube bundle keeps increasing along the flow direction and the crystallization range spreads from the tube wall to the center of the tube bundle;
6. The region of easy crystallization was determined to be the vortex core area with a lower temperature, higher velocity and greater gas phase content, located in the area enclosed by the top of the first pipe side and the bottom of the second pipe side in the vertical flow direction between 0.1−0.4 m. Those are the two sides of the center of the tube. It is determined that crystallization occurs first in this area, and that the amount of crystallization is relatively large. The maximum mass fraction of ammonium chloride is 1.16 × 10^−4^;
7. The corrosion failure forms caused by ammonium chloride in the area of easy crystallization are under-deposit corrosion and acid corrosion. It can be seen from the actual corrosion failure morphology that under-deposit corrosion occurs in the heat exchanger. When the ammonium chloride is diluted in water, a high concentration of the ammonium chloride solution is formed, which causes the acid corrosion.

## Figures and Tables

**Table 1:** Composition of scaling material.

Element	ω, %	x, %
N	22.89	40.86
O	5.08	7.94
Al	6.71	6.21
S	0.83	0.65
Cl	60.05	42.34
Fe	4.44	1.99

**Table 2:** Mass flow of the inlet.

	Gas Phase	Oil Phase
mass flow (kg/s)	3.6225	104.1667

**Table 3:** Gas phase component content at inlet.

The Gas Phase Composition	The Mole Fraction of Each Component
NH_3_	0.001844221
HCl	7.667234 × 10^−6^
H_2_	0.9981484108

**Table 4:** Gas phase component content at inlet.

Multiphase Flow Component	Specific Heat Capacity (J/kg·K)	Mass Flow (kg/s)
H_2_	14,180.6	3.312
NH_3_	2303.1	0.05198
HCl	988.9	0.0004644
Oil phase	2135	104.2
Water phsae	4200	6.944

## Data Availability

The data presented in this study are available upon request.

## References

[1] Groysman A. (2017). Corrosion problems and solutions in oil, gas, refining and petrochemical industry. Koroze A Ochr. Mater..

[2] Akpanyung K.V., Loto R.T., Fajobi M.A. (2019). An Overview of Ammonium Chloride (NH_4_Cl) Corrosion in the Refining Unit. J. Phys. Conf. Ser..

[3] Ossai C.I., Boswell B., Davies I.J. (2015). Pipeline failures in corrosive environments—A conceptual analysis of trends and effects. Eng. Fail. Anal..

[4] Liu X., Zhu H., Yu C., Jin H., Wang C., Ou G. (2021). Analysis on the corrosion failure of U-tube heat exchanger in hydrogenation unit. Eng. Fail. Anal..

[5] Zhu M., Ou G., Jin H., Wang K., Zheng Z. (2015). Top of the REAC tube corrosion induced by under deposit corrosion of ammonium chloride and erosion corrosion. Eng. Fail. Anal..

[6] Wang K. (2014). Prediction of ammonium salt crystallization deposition and study on corrosion law in petrochemical system. Master’s Thesis.

[7] Toba K., Ueyama M., Kawano K., Sakai J. (2012). Corrosion of Carbon Steel and Alloys in Concentrated Ammonium Chloride Solutions. Corrosion.

[8] Wang H., Li Y., Cheng G., Wu W., Zhang Y., Li X. (2018). Electrochemical Investigation of Corrosion of Mild Steel in NH_4_Cl Solution. Int. J. Electrochem. Sci..

[9] Wang H., Li Y., Cheng G., Wu W., Zhang Y.-H. (2018). A Study on the Corrosion Behavior of Carbon Steel Exposed to a H_2_S-Containing NH_4_Cl Medium. J. Mater. Eng. Perform..

[10] Laitinen T. (2000). Localized corrosion of stainless steel in chloride, sulfate and thiosulfate containing environments. Corros. Sci..

[11] Baranwal P.K., Rajaraman P.V. (2019). Electrochemical investigation on effect of sodium thiosulfate (Na_2_S_2_O_3_) and ammonium chloride (NH_4_Cl) on carbon steel corrosion. J. Mater. Res. Technol..

[12] Baranwal P.K., Venkatesh R.P. (2017). Investigation of carbon steel anodic dissolution in ammonium chloride solutions using electrochemical impedance spectroscopy. J. Solid State Electrochem..

[13] Ou G., Wang K., Zhan J., Tang M., Liu H., Jin H. (2013). Failure analysis of a reactor effluent air cooler. Eng. Fail. Anal..

[14] Zheng Z., Ou G., Ye H., Tan J., Jin H. (2017). Analysis on the under deposit corrosion of air cooler tubes: Thermodynamic, numerical and experimental study. Eng. Fail. Anal..

[15] Ou G., Jin H., Xie H., Cao J., Qiu J. (2011). Prediction of ammonium salt deposition in hydroprocessing air cooler tubes. Eng. Fail. Anal..

[16] Jin H., Chen X., Ou G., Zhang J. (2020). Potential failure analysis and prediction of multiphase flow corrosion thinning behavior in the reaction effluent air cooler system. Eng. Fail. Anal..

[17] Tang P., Yang J., Zheng J., Wong I., He S., Ye J., Ou G. (2009). Failure analysis and prediction of pipes due to the interaction between multiphase flow and structure. Eng. Fail. Anal..

[18] El-Gammal M., Mazhar H., Cotton J.S., Shefski C., Pietralik J., Ching C.Y. (2010). The hydrodynamic effects of single-phase flow on flow accelerated corrosion in a 90-degree elbow. Nucl. Eng. Des..

[19] Mohammadi F., Luo J. (2010). Effects of particle angular velocity and friction force on erosion enhanced corrosion of 304 stainless steel. Corros. Sci..

[20] Wu Y. (1994). Calculations estimate process stream depositions. Oil Gas J..

[21] Sun A., Fan D. (2010). Prediction, Monitoring, and Control of Ammonium Chloride Corrosion in Refining Processes. Proceedings of the Corrosion Conference and Expo 2010.

[22] Sliem M.H., Fayyad E.M., Abdullah A.M., Younan N.A., Arora D. (2021). Monitoring of Under deposit Corrosion for the Oil and Gas Industry: A Review. J. Pet. Sci. Eng..

[23] Liu X., Duan A., Quan J., Jin H., Wang C. (2020). A study on the corrosion failure induced by the ammonium chloride deposition in a high-pressure air cooler system. Eng. Fail. Anal..

